# Supplementary material for: Understanding the Intrinsic and Extrinsic Motivations Associated with Community Gardening to Improve Environmental Public Health Prevention and Intervention
Source: Int J Environ Res Public Health. 2019 Feb 11;16(3):494. doi: 10.3390/ijerph16030494 (PMC6388228; doi:10.3390/ijerph16030494)
Supplement: Supplementary file 1 [file ijerph-16-00494-s001.pdf]

Supplemental Material

Understanding the Intrinsic and Extrinsic Motivations Associated with Community Gardening to Improve Environmental Public Health Prevention and Intervention

Monica D. Ramirez-Andreotta, Abigail Tapper, Diamond Clough, Jennifer S. Carrera and Shana Sandhaus

Supplemental Material

<u>**Conducting the interview:**</u>

- *Greet and thank the participant for their time and succinctly introduce the project.*
  - State that purpose of the research project is to learn more about people's gardening experiences and behavior. By learning about your experiences, we will be able to identify environmental health research gaps, ways to improve the quality of urban gardens, the type of support and infrastructure that gardeners need, and other items that can improve one's urban garden experience. For more information, please refer to the informed consent form.

- *Give them the informed consent form*
  - Briefly summarize: the interview is voluntary; they may withdraw from the study at any time or skip any question they don't want to answer. We want to record the interview, because we want to thoroughly capture their comments, is that ok?

- *Give them an opportunity to read the consent and share any questions or concerns they have before they sign.*
  - Once you have answered all their questions and they've signed and dated the consent form, ask them if they would like a copy of the form they signed for their records and provide them with a copy if they say yes.

- *Turn on both digital voice recorders and point the microphones towards the participant.*

- *Begin recording.*

- *Thank the participant again for agreeing to participate in the interview.*

- *Ask the remaining participant questions on the Interview Cover Sheet (7-10)*

<u>**Garden Interview Questions**</u>

1. What led you to gardening? Why do you garden?

2. How long have you been gardening?

3. How did you learn how to garden?

4. Do you garden at a community garden? If so, which one? How did you get involved in community gardens?

5. How long have you been gardening at the community garden in your neighborhood?

6. Do you also have a home garden? [IF YES] Which one do you garden in most? Why?

7. What has been your experience so far at the community garden?

8. On average, how long are you in the community garden, per day, per week?
9. How does this compare with the time you spend in your own, home garden?

10. On average, how much do you eat from their garden— per day, per week? Who else eats from your garden or garden plot? Who do you share with?

11. What do you typically grow? How do you choose what seeds to buy? Where or how do you get your seeds?

12. What is your gardening routine?
    a. What protective gear, if any, do you wear (e.g., gloves, hats, long sleeves)?
    b. Do you wash your hands after working?
    c. How do you wash your vegetables?

13. What health concerns do you have about store bought vegetables? Where do you shop for your vegetables?

14. What health concerns do you have about garden grown vegetables?

15. Are there any environmental or health issues neighboring your garden or in your neighborhood?

16. Are there questions you would like to have studied about soil or water quality in your garden?

17. Have you done any soil or water testing on your land or where you are currently gardening?

18. How do you assess your soil and vegetable quality? [If unsure of question] Prompt with examples like: manuals, tools, soil testing kits, water testing, PH testing?

19. Do you know about the historical uses of the garden space? Do you know was there before it was a garden?

[IF YES] How did you learn about it?
[IF NO] Do you want more information about it?

20. Who is responsible for the safety of the soil and vegetables being grown in your garden? Or who should be?

21. Have you encountered any challenges while gardening in an urban space?
    a. [IF YES] How did you solve them?
    b. What kinds of creative fixes have you come up with to improve your garden?

22. Have you implemented any strategies in your garden to get rid of unwanted soil, water or waste?
    a. [IF YES] Ask them to describe.

23. What would you like to change at your garden?

24. What have you learned through gardening?

25. What benefits have you received from gardening? [If unsure of question, prompt with examples like: new friends, activities, food sharing?]

26. Do you consider gardening a form of exercise? How does this exercise help (or hurt) your body?

27. How do you get to your community garden?

28. What are your thoughts regarding the health benefits from gardening?

29. Would you like there to be more activities at your garden?

30. [IF YES] What type of activities would you want?
    What are best ways to communicate with you regarding gardening issues? Are there certain communication strategies that you recommend?

31. Do you have anything else you would like to add?

<u>Finally, I just have a couple of basic background questions about you.</u>

1. How would you describe your gender? ___________________________
2. How would you describe your race/ethnicity? _____________________
3. What is your zip code? _________
4. How old are you? ___________

Thanks so much. Let me get your gift bag.

<u>At the end of the interview:</u>

1. When the interview is finished, turn off both tape recorders.

2. Thank the participant for their time.

3. Give the participant the gift bag.

4. Make sure you wrote the voice recorder file number on the Interview Cover Sheet.

5. Copy the sound file to the appropriate folder in the external hard drive connected to the laptop. Each file needs to be given the Garden Project ID number.
